# Comparison of the performances of low-crystalline carbonate apatite and Bio-Oss in sinus augmentation using three-dimensional image analysis

**DOI:** 10.1186/s40729-021-00303-4

**Published:** 2021-03-23

**Authors:** Koudai Nagata, Kei Fuchigami, Ryoji Kitami, Yurie Okuhama, Kana Wakamori, Hirokazu Sumitomo, Hyunjin Kim, Manabu Okubo, Hiromasa Kawana

**Affiliations:** Department of Oral and Maxillofacial Implantology, Kanagawa Dental University, 82 Inaoka-cho, Yokosuka, 238-8580 Japan

**Keywords:** CBCT, Cone-beam computed tomography, Maxillary sinus augmentation, Dental implant, Sinus lift, Bio-Oss®, Cytrans®

## Abstract

**Background:**

In locations where the alveolar bone height is low, such as at the maxillary molars, implant placement can be difficult, or even impossible, without procedures aimed at generating new bone, such as sinus lifts. Various types of bone graft materials are used after a sinus lift. In our study, a three-dimensional image analysis using a volume analyzer was performed to measure and compare the volume of demineralized bovine bone mineral (Bio-Oss®) and carbonate apatite (Cytrans®) after a sinus lift, as well as the amount of bone graft material resorption. Patient data were collected from cone-beam computed tomography images taken before, immediately following, and 6 months after the sinus lift. Using these images, both the volume and amount of resorption of each bone graft material were measured using a three-dimensional image analysis system.

**Results:**

The amount of bone resorption in the Bio-Oss®-treated group was 25.2%, whereas that of the Cytrans®-treated group was 14.2%. A significant difference was found between the two groups (*P* < 0.001).

**Conclusions:**

Our findings indicate that the volume of bone resorption was smaller in the Cytrans®-treated group than in the Bio-Oss®-treated group, suggesting that Cytrans® is more promising for successful implant treatments requiring a sinus lift.

## Background

Dental implant therapy has advanced significantly over the last decade. Currently, implants are available in various sizes and surface textures to provide patients with improved implant therapy options [1–3]. However, at sites in the upper molar region, the alveolar bone height is less than that at other sites, and the effects of alveolar bone resorption after tooth extractions should also be considered. Therefore, implant placement may be difficult, or impossible, without bone augmentation procedures, such as a sinus lift [4–6]. While the sinus lift procedure is an established treatment option [7–9], the most suitable choice of bone-filling material is unclear, and novel bone-filling materials are currently under development [10–12]. In this study, we evaluate the utility of demineralized bovine bone mineral (DBBM), commercially known as Bio-Oss®, and carbonate apatite (CO_3_Ap), commercially known as Cytrans®, as bone-filling materials in a sinus lift. We conducted a comparative study by measuring the volume and the amount of resorption of each material using a three-dimensional image analysis system.

## Methods

### Patients

This study was conducted on patients who elected to receive dental implant therapy requiring a sinus lift between January 2019 and May 2020 (12 individuals, mean age 58.2 years). The sinus lift sites examined in this study consisted of a total of 14, with 8 sites included in the DBBM-treated (Bio-Oss®) group and 6 sites in the CO_3_Ap-treated (Cytrans®) group.

These procedures were carried out once the study parameters were explained to the patients, and they gave their consent. This study was approved by the Kanagawa Dental University Ethics Committee (approval number 697). The following were the inclusion criteria for the subjects: age of at least 20 years old, free of systemic diseases, non-smokers, with bone height of less than 3 mm, and with no thickening of the sinus mucosa.

Before performing the sinus lift, patients whose teeth still remained were treated for periodontal disease; if there was decay in the teeth adjacent to the defect, the decay was treated, and if there was a root canal lesion, root canal therapy was performed to reduce the risk of surgery.

### Surgical procedure

All patients were instructed to take an oral dose (1 g) of amoxicillin hydrate (Sawacillin Capsules®, LTL Pharma, Tokyo, Japan) 1 h before surgery. Infiltration anesthesia was administered (Lidocaine/Adrenaline bitartrate®, Showa Yakuhin Kako Co., Ltd., Tokyo, Japan), a gingival incision was made, and avulsion was performed up to the sidewall of the maxillary sinus. The Schneiderian membrane was elevated using the lateral window technique, and bone-filling was performed using either DBBM (Bio-Oss®, Geistlich, Wolhusen, Switzerland) or CO_3_Ap (Cytrans®, GC, Tokyo, Japan).

Finally, the fenestration site was covered with a collagen membrane (Bio-Gide®, Geistlich, Wolhusen, Switzerland) and sutured. All surgical procedures were performed in two stages. The stitches were removed 2 weeks after surgery.

All surgeries were performed by the same doctor, a teaching associate in the Department of Implantology at our university hospital. The transplant material was also infiltrated with saline solution.

Cytrans (size, M; particle size, 0.6–1.0 mm) and Bio-Oss (size, L; particle size, 1–2 mm) were the grafting materials used.

### Volume evaluation method

**Fig. 1:**
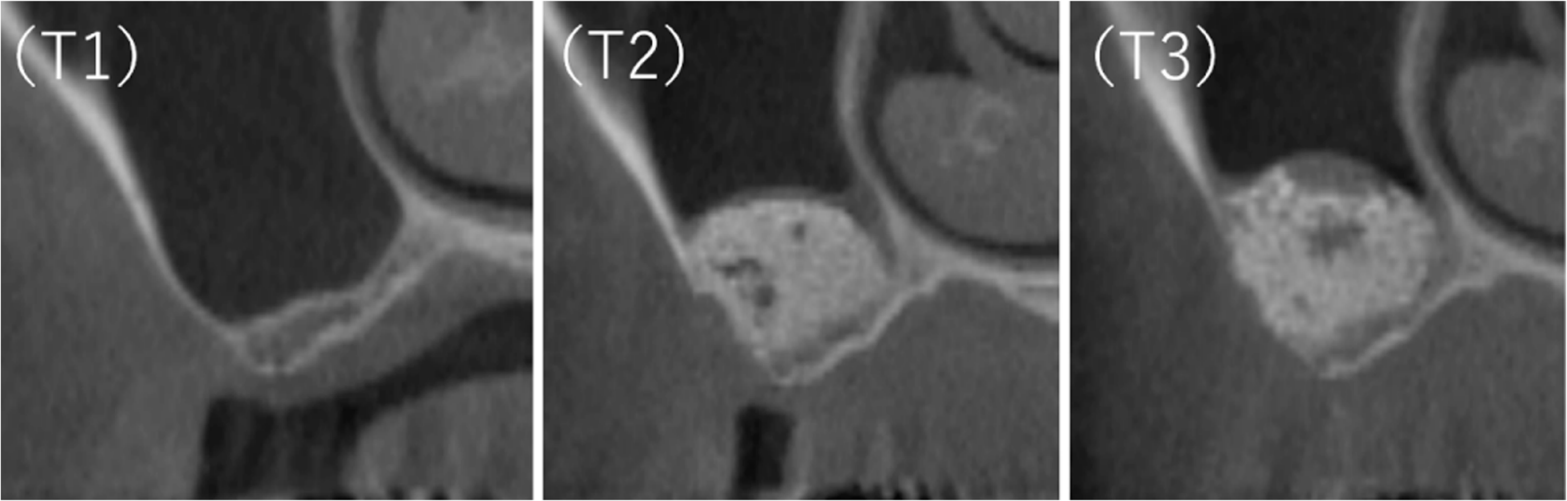
CBCT images of the maxillary sinus: (T1) before surgery, (T2) immediately after surgery, and (T3) 6 months after surgery

Evaluations were conducted using patient data obtained from cone beam computed tomography (CBCT) images (3DX®, Morita, Tokyo, Japan) collected before (T1), immediately following the sinus lift surgery (T2), and 6 months later (T3) (Fig. 1).

The collected images were superimposed using a three-dimensional image analysis system volume analyzer (SYNAPSE VINCENT®, FUJIFILM, Tokyo, Japan), and the volume of bone-filling material was measured. Volumes were quantified according to the following method: Using the fusion function of the three-dimensional (3D) visualization software SYNAPSE VINCENT®, the volume immediately after surgery was measured by overlaying T1 and T2 images, and the volume at 6 months after surgery was measured by overlaying T1 and T3 images. The amount of resorption was calculated by subtracting the volume on T3 images from that on T2 images.

**Fig. 2:**
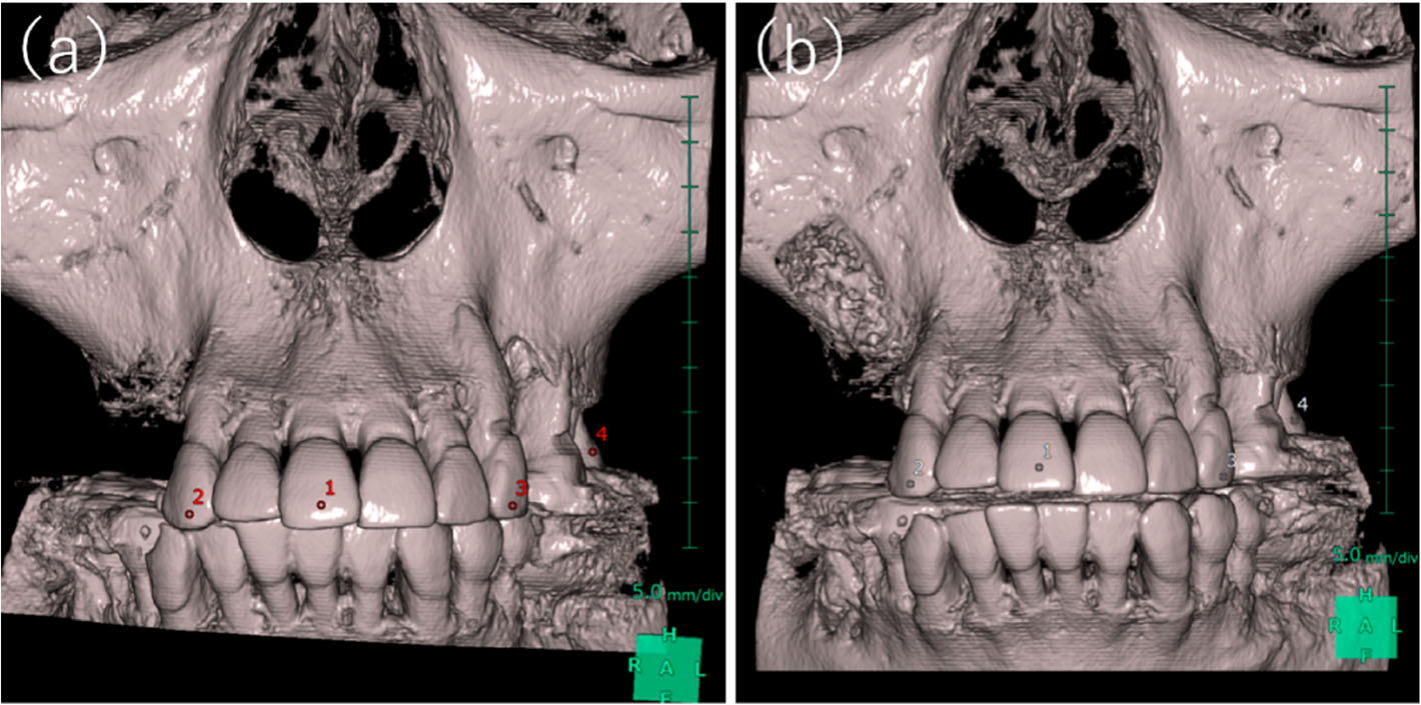
“Manual alignment” was performed using residual teeth as reference points. **a** Preoperative CBCT data. **b** Postoperative CBCT data

**Fig. 3:**
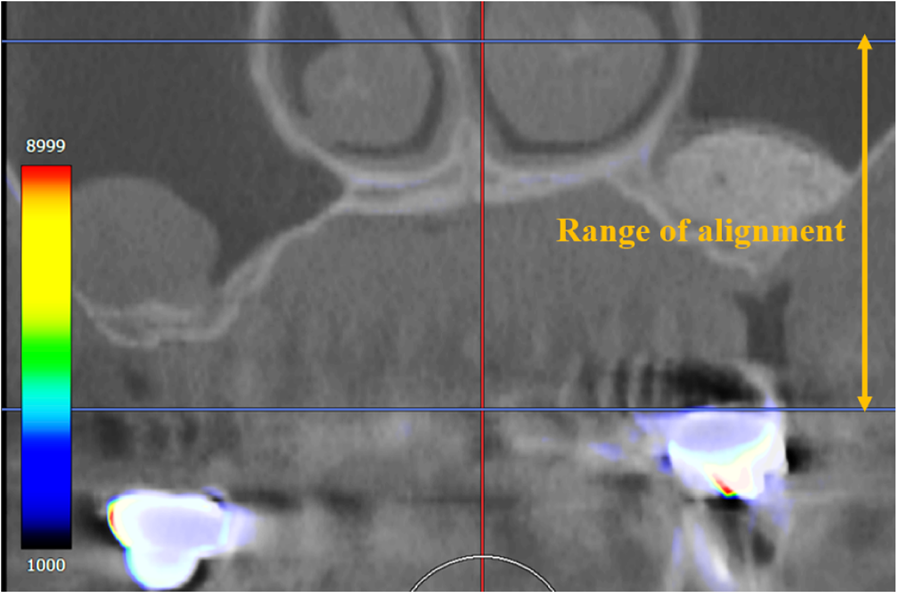
“Automatic alignment” was performed using maxillary arch anatomical landmarks as reference points

**Fig. 4:**
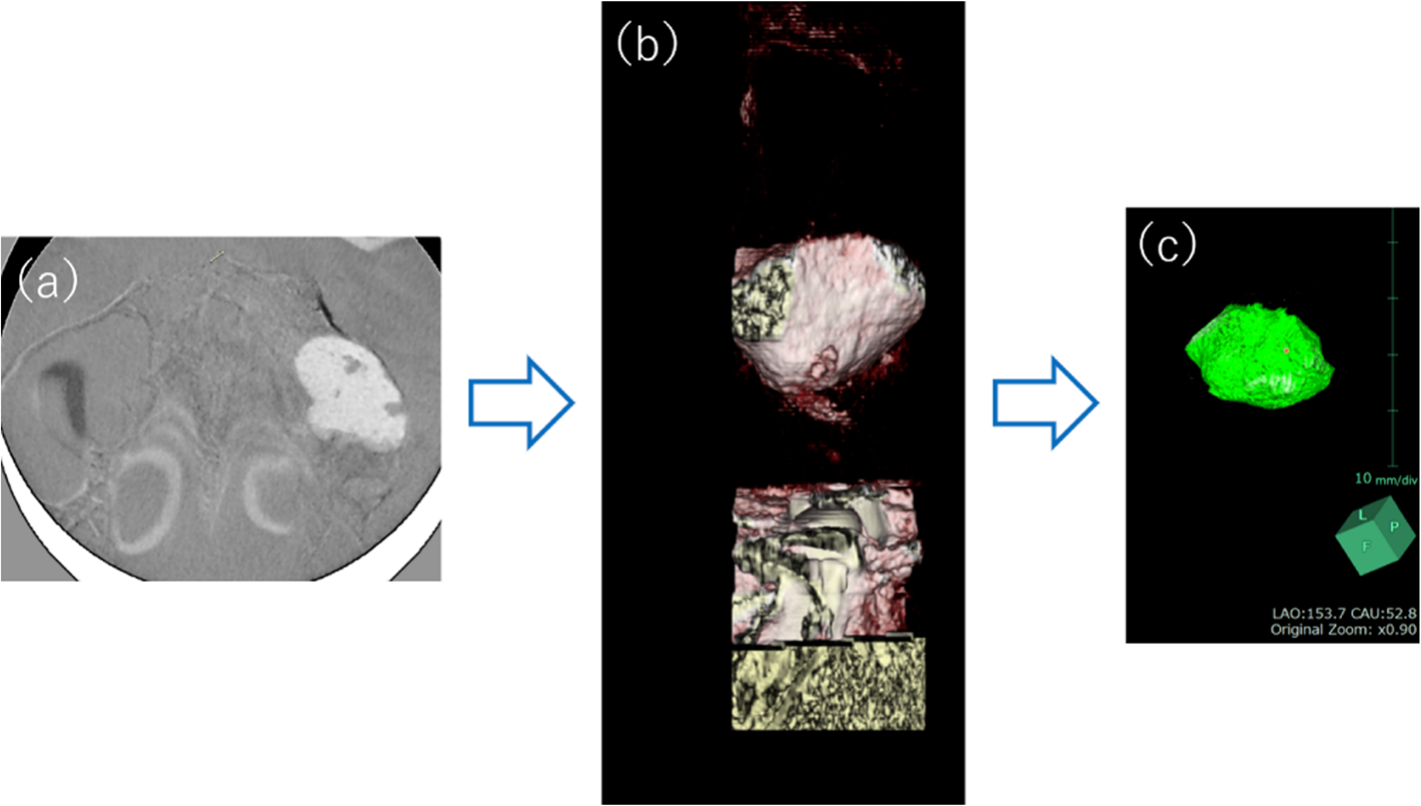
The overlaid images were trimmed, and the volume was measured using “overall measurements.” Image obtained after **a** “image reconstruction,” **b** trimming on the vertical axis, and **c** trimming of unnecessary data (information)

Specifically, the overlay of images involved the import of the two images into SYNAPSE VINCENT®, followed by a “manual image alignment” (Fig. 2) and an “automatic image alignment” using maxillary arch anatomical landmarks as references (Fig. 3). Finally, the overlay of processed images was produced. Next, an “image reconstruction” step was performed, followed by the removal of excess information by trimming the resulting 3D data, and “overall measurement” was selected among the measurement methods available to obtain the volume of the bone-filling material (Fig. 4).

### Statistical methods

The comparison of the amount of resorption in the context of each bone-filling material was performed using the Student’s *t*-test (*P* < 0.05).

## Results

**Fig. 5:**
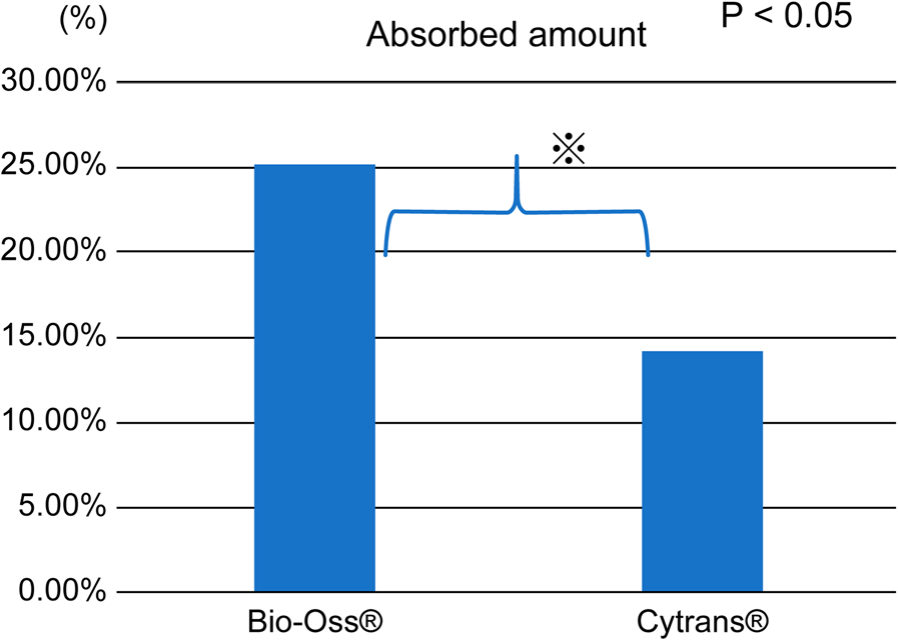
Amount of resorption of Bio-Oss® and Cytrans®

In the Bio-Oss®-treated group, bone resorption was 25.2%, whereas in the Cytrans®-treated group, bone resorption was 14.2%. There was a significant difference in bone resorption between the two bone-filling materials (*P* = 0.001) (Fig. 5) (Table 1). In addition, there was no observed increase in bone mass.

**Table 1:** Clinical data on the volume of bone-filling material in procedures using Bio-Oss® and Cytrans®

Treatment group	Patient no.	Age	Gender	Deficit condition (FDI)	T2 (ml)	T3 (ml)	Absorbed amount (%)
Bio-Oss® (*n* = 8)	1	65	F	Edentulous	3.762	2.538	32.50
	2	59	M	23–27	1.583	1.167	26.30
	3	53	M	26, 27	2.271	1.772	22.00
	4	59	F	14–17	4.880	3.836	21.40
	5	21	F	14–16	1.428	0.916	35.90
	6	70	F	26, 27	2.163	1.622	25.00
	7	71	F	Edentulous	2.436	1.973	19.00
	8	71	F	Edentulous	3.198	2.581	19.30
Cytrans® (*n* = 6)	1	67	F	14–17	2.547	2.371	6.90
	2	41	F	16, 17	2.108	1.791	15.00
	3	59	M	25–27	1.506	1.222	18.90
	4	67	M	Edentulous	3.763	3.375	10.30
	5	67	M	Edentulous	2.392	2.150	10.10
	6	67	F	24–27	2.780	2.386	14.20

Postoperatively, most patients developed swelling on the second day, followed by slight internal bleeding on the cheek in some cases. The internal bleeding disappeared in about 2 weeks.

All patients showed a good postoperative prognosis and were able to reach 6 months postoperatively without any problems.

## Discussion

Several reports have examined the changes that occur in bone replacement materials after a sinus lift. Our findings suggest that the amount of resorption after surgery could be reduced by using Cytrans® as the bone-filling material.

Various methods have been used to measure the changes in the volume of bone-filling materials after a sinus lift. In a study conducted on 27 participants, Kim et al. used preoperative and postoperative panoramic X-ray images to measure and compare the distance of implants placed simultaneously at three points: mesial, central, and distal. They reported that in all cases, bone resorption occurred over time [13]. Although the avoidance of CBCT imaging may be useful in the evaluation of ongoing follow-up treatment because of the reduced exposure of patients to radiation [14, 15], a complete picture of the anatomical morphology is difficult to obtain using 2D images alone compared with panoramic x-rays and CBCT data [16–18].

Gorla et al. quantified the volume of bone-filling material after a sinus lift using CBCT by measuring the cross-sectional area and height at randomly selected locations [19]; however, in recent years, three-dimensional measurements using CBCT have been made possible. Previously, Kwon et al. conducted sinus lifts using DBBM (Bio-Oss®) and measured the changes in bone-filling volume using a method similar to ours and reported that the volume was fully resorbed in 2 to 26 weeks after surgery [20]. In a similar study using DBBM (Bio-Oss®), Younes et al. reported that the volume of the graft was 1418.26 mm^3^ 2 weeks after surgery, 1201.21 mm^3^ 3 months after surgery, and 1130.13 mm^3^ 2 years after surgery, and that the stability of the bone-filling materials was 79.7% [21].

Previous studies have reported favorable DBBM (Bio-Oss®) bone resorption values after a sinus lift, including 19.4% according to Guo et al., 9.39 ± 3.01% according to Gultekin et al., and 26% according to Kirmeier et al. [22–24]. Thus, sinus lifts using DBBM (Bio-Oss®) as the bone-filling material typically yield positive results. It is often debated whether DBBM (Bio-Oss®) should be mixed with autologous bone during bone transplantation. In a systematic review of sinus lift procedures, Rickert et al*.* concluded that artificial bone substitutes should be mixed with autologous bone to promote bone formation [25]. A systematic review by Aludden et al. revealed similar results [26]. Furthermore, Hatano et al. previously reported positive results using graft materials made of a mixture of Bio-Oss® and autologous bone at a ratio of 2:1 [27]. However, in experiments conducted by Kim et al., no difference in new bone formation was observed, regardless of whether the graft material was composed of Bio-Oss® alone or made of Bio-Oss® mixed with 25% of autologous bone [28]. A systematic review published by Jensen et al. described analogous results [29]. Similarly, Starch-Jensen et al. reported that the long-term prognosis after a sinus lift was favorable, regardless of the filling material used [30]. Mixing DBBM (Bio-Oss®) with an autologous bone is believed to promote bone formation; however, the optimal method to collect sufficient amounts of autologous bone remains unclear.

Clinical reports using Cytrans® are few; however, Kudoh et al. used this material to perform sinus lifts in humans with good results [31]. Ishikawa et al. and Mano et al. compared the osteogenic potential of three different types of synthetic bones (Neobone®, Cytrans®, and Cerasorb®) in dogs and reported that the degree of bone replacement was the largest using Cytrans® [32, 33]_._ Additionally, Fujisawa et al. reported that the porosity and carbonate content of Cytrans® were 25.4 ± 0.6% and 12.1 ± 0.6%, respectively, whereas those of Bio-Oss® were 57.0 ± 0.5% and 5.6 ± 0.1%, respectively.

The low crystallinity, porous structure, and high porosity of Bio-Oss® make it desirable for the replacement of osteoconductive bone. In contrast, Cytrans, with a higher carbonate content, led to a faster rate of bone formation and a higher amount of new bone formation. However, the justification of these differences in bone formation is yet unknown [34]. Of note, Spence et al. reported that a high carbonate content can increase osteoclastogenesis in the context of carbonate-substituted hydroxyapatite [35]; increased osteoclastogenesis leads to the activation of osteoblasts. Therefore, we hypothesize that the potential increased osteoclastogenesis in the context of carbonate-rich Cytrans leads to an increase in the rate of bone formation.

In this study, the amount of resorption in the Cytrans® group was low. As reported above, Cytrans® has a high carbonate content and a high rate of replacement in new bone, which may have resulted in less postoperative resorption.

Chan et al. classified the maxillary sinus floor as follows: narrow, average, and wide, and reported that the narrow floor makes the lateral approach technique more difficult [36]. Cho et al. reported that the angular difference between the medial and lateral walls of the maxillary sinus makes surgery difficult and increases the likelihood of the perforation of the Schneider’s membrane [37]. The presence of a septum in the maxillary sinus is also thought to be one of the factors that makes surgery difficult [38]. However, in this surgery, there was no septum and no perforation of the Schneider membrane; thus, the volume was not affected.

The limitation of this study is that although we could visually confirm the change of the graft material from sparse to dense on the 3D visualization software, it was impossible to evaluate the density based on the CT values because of the CBCT imaging. Pauwels et al. compared CBCT with multidetector CT and reported that CBCT produces a large variability in gray values due to its limited field size, relatively large amount of scattered radiation, and limitations of currently applied reconstruction algorithms. Of note, they reported that CBCT should not be used to assess bone quality and density [39]. In addition, it was not possible to calculate the bone resorption and survival rate from the time of superstructure attachment; this represents an area of focus for future research.

## Conclusion

Our study findings revealed that the amount of volume resorption was smaller when Cytrans® was used as a bone-filling material than when Bio-Oss® was used. This study provides a potential framework for improved dental implant treatment outcomes in the future.

## Data Availability

The datasets obtained and analyzed during the current study are available from the corresponding author upon reasonable request.

## Abbreviations

3D: Three-dimensional
CBCT: Cone beam computed tomography
DBBM: Demineralized bovine bone mineral
CO_3_Ap: Carbonate apatite
